# Use of Hydrogel Electrolyte in Zn-MnO_2_ Rechargeable Batteries: Characterization of Safety, Performance, and Cu^2+^ Ion Diffusion

**DOI:** 10.3390/polym16050658

**Published:** 2024-02-28

**Authors:** Jungsang Cho, Damon E. Turney, Gautam Ganapati Yadav, Michael Nyce, Bryan R. Wygant, Timothy N. Lambert, Sanjoy Banerjee

**Affiliations:** 1 The CUNY Energy Institute, City University of New York, 160 Convent Ave, New York, NY 10031, USA; chojs0114@gmail.com (J.C.); mnyce1957@gmail.com (M.N.); sanjoy@urbanelectricpower.com (S.B.); 2 Urban Electric Power, Pearl River, NY 10965, USA; gautam@urbanelectricpower.com; 3 Sandia National Laboratories, Department of Photovoltaics and Materials Technology, Albuquerque, NM 87185, USA; bwygant@sandia.gov (B.R.W.); tnlambe@sandia.gov (T.N.L.); 4 Sandia National Laboratories, Center of Integrated Nanotechnologies, Albuquerque, NM 87185, USA

**Keywords:** hydrogel, zinc, manganese dioxide, rechargeable, diffusion, energy storage

## Abstract

Achieving commercially acceptable Zn-MnO_2_ rechargeable batteries depends on the reversibility of active zinc and manganese materials, and avoiding side reactions during the second electron reaction of MnO_2_. Typically, liquid electrolytes such as potassium hydroxide (KOH) are used for Zn-MnO_2_ rechargeable batteries. However, it is known that using liquid electrolytes causes the formation of electrochemically inactive materials, such as precipitation Mn_3_O_4_ or ZnMn_2_O_4_ resulting from the uncontrollable reaction of Mn^3+^ dissolved species with zincate ions. In this paper, hydrogel electrolytes are tested for MnO_2_ electrodes undergoing two-electron cycling. Improved cell safety is achieved because the hydrogel electrolyte is non-spillable, according to standards from the US Department of Transportation (DOT). The cycling of “half cells” with advanced-formulation MnO_2_ cathodes paired with commercial NiOOH electrodes is tested with hydrogel and a normal electrolyte, to detect changes to the zincate crossover and reaction from anode to cathode. These half cells achieved ≥700 cycles with 99% coulombic efficiency and 63% energy efficiency at C/3 rates based on the second electron capacity of MnO_2_. Other cycling tests with “full cells” of Zn anodes with the same MnO_2_ cathodes achieved ~300 cycles until reaching 50% capacity fade, a comparable performance to cells using liquid electrolyte. Electrodes dissected after cycling showed that the liquid electrolyte allowed Cu ions to migrate more than the hydrogel electrolyte. However, measurements of the Cu diffusion coefficient showed no difference between liquid and gel electrolytes; thus, it was hypothesized that the gel electrolytes reduced the occurrence of Cu short circuits by either (a) reducing electrode physical contact to the separator or (b) reducing electro-convective electrolyte transport that may be as important as diffusive transport.

## 1. Introduction

Zinc (Zn)–manganese dioxide (MnO_2_) rechargeable batteries have drawn research interest because of their safe, affordable, and environmentally friendly properties. Further, both Zn and MnO_2_ have high theoretical specific capacities of 820 mAh/g and 617 mAh/g, respectively, which creates an opportunity for a commercially feasible battery. The high capacity of MnO_2_ is predicated on it hosting two-electron reaction chemistry. Aqueous potassium hydroxide (KOH) has facilitated these electrochemical reactions in MnO_2_ rechargeable batteries [1,2,3,4], shown to be a first electron reaction involving a proton insertion reaction and a second electron reaction involving an MnO_2_ dissolution—precipitation reaction. For the anode, Zn undergoes dissolution on discharge and precipitation on charge, as described in Equations (1)–(4):

Zn anode:

(1) $$\;\mathrm{Zn}+4{\mathrm{OH}}^{-}\;↔\;\mathrm{Zn}{\left(\mathrm{OH}\right)}_{4}^{2-}+2e^{-}$$

(2) $$\mathrm{Zn}{\left(\mathrm{OH}\right)}_{4}^{2-}\;↔\;\mathrm{ZnO}+2{\mathrm{OH}}^{-}+H_{2}O\;\;\;\;\;\;\;\;\;\mathrm{direct}\;\mathrm{dissolution}–\mathrm{precipitation}\;$$

MnO_2_ cathode:

(3) $${\mathrm{MnO}}_{2}+H_{2}O+e^{-}\;\to \mathrm{MnOOH}+{\mathrm{OH}}^{-}\;\;\;\;\;\;\;\;\;\;\;\;\;\mathrm{proton}\;\mathrm{insertion}\;\mathrm{reaction}\;$$

(4) $$\mathrm{MnOOH}+H_{2}O+e^{-}\;\to \mathrm{Mn}{\left(\mathrm{OH}\right)}_{2}+{\mathrm{OH}}^{-}\;\;\;\;\;\;\;\;\;\mathrm{dissolution}–\mathrm{precipitation}\;\mathrm{reaction}$$

It is hypothesized that using liquid electrolytes can exacerbate battery failure. Mn ions dissolve during the second electron reaction process, which can lead to Mn loss due to migration and diffusion to distant sites, hypothetically accelerating the formation of inactive phases like spinel hausmannite (Mn_3_O_4_) when [Mn-OH] complexes react with each other or re-precipitate at non-local sites, all resulting in the loss of active Mn ions. At the Zn electrode, liquid electrolytes amplify Zn redistribution, which can lead to Zn electrode shape change and passivation, in turn leading to pore plugging and uncontrollable redeposition of Zn during charge at high current densities and dendrite formation [5,6]. Also, liquid electrolytes used for Zn-MnO_2_ rechargeable batteries have lower viscosity (relative to gelled electrolytes) and therefore leak easily through cracks in the battery housing, if damaged, thereby creating safety issues for battery transportation. The previous literature [7,8,9,10,11,12] found that copper (Cu) and bismuth (Bi) helped Mn reversibility, accessing the second electron reaction region with long battery cycle life. Compared to the recent research [13,14], our group’s method of electrode fabrication [9,10] is easier than electrodeposition, and the cycling performance paired with Zn achieved a longer cycle life. We thus denote this electrode fabrication as “advanced” compared to previous methods. However, failure mechanisms during the second electron reaction region were also reported, specifically, that if zincate ions diffuse across the separator into the cathode region, they can react with dissolved Mn^3+^ ions to form hetaerolite (ZnMn_2_O_4_), which is electrochemically inert. This results in the loss of active Mn^3+^ ions, and battery performance is directly affected by the reversibility of Zn and MnO_2_ during the dissolution–precipitation reaction, as shown in Equations (1)–(4).

Our group previously reported that gel electrolytes can mitigate failure mechanisms for Zn-MnO_2_ batteries constrained to just the first electron reaction of MnO_2_ [15]. Gel electrolytes have been under research for beneficial properties between solid and liquid electrolytes, with improved safety, yet retaining the self-healing property of liquid electrolyte [16,17,18,19]. We showed that the gel electrolyte reduced the migration of zincate ions (Zn(OH)_4_^2−^), suggesting that the formation of the electrochemically inactive material, hetaerolite (ZnMn_2_O_4_), was mitigated. Moreover, we found that gel electrolyte reduced dissolution of each active material. Recent studies containing hydrogels, which are chemically similar to the hydrogel reported in this manuscript, have demonstrated their ability to enhance the performance of zinc-based batteries when compared to the use of liquid KOH electrolytes [20,21,22]. However, the battery chemistry referenced in Ref. [20] is based on zinc–nickel, which typically yields an energy density of approximately 140 Wh L^−1^. This is lower than that of Zn-MnO_2_ batteries, which can reach >400 Wh L^−1^. In the cases of Refs. [21,22], Zn-MnO_2_ battery chemistry was utilized. However, the results obtained were cycled in the first electron reaction of MnO_2_, suggesting that higher energy achievement can be made by accessing the second electron reaction of MnO_2_.

Here, we expand those experiments into the second electron reaction of MnO_2_ where higher cathode capacity is accessed. We also report on our safety analysis of the liquid vs. hydrogel electrolytes, via U.S. Department of Transportation (DOT) standards that define a battery to be non-spillable if the electrolyte does not flow from a rupture or crack in the battery case [23]. To develop highly energy-dense batteries for the second electron reaction technology of Zn-MnO_2_ batteries with gel electrolytes, we present battery cycling results under second electron reaction chemistry, along with ion diffusion properties in hydrogel electrolytes.

## 2. Materials and Methods

### 2.1. Hydrogel Synthesis

Hydrogel electrolytes, specifically potassium polyacrylate gels, were synthesized with liquid KOH electrolytes, acrylic acid (AA, Sigma Aldrich, St. Louis, MO, USA), *N*,*N*′-methylenebisacrylamide (MBA, cross-linker, Sigma Aldrich), and potassium persulfate (K_2_S_2_O_8_, initiator, Sigma Aldrich). The final pH of the hydrogel electrolyte was ensured to be ~25 wt.% KOH. The synthesis process is the same as reported in Ref. [15]. Liquid KOH electrolytes were made with KOH pellets purchased from Fisher Scientific, and the AA, MBA, and K_2_S_2_O_8_ were purchased from Sigma Aldrich. All components were used without further treatment. The mole fraction composition of the hydrogel was 1:0.156:0.0484:4.096 × 10^−6^ in terms of H_2_O:KOH:Acrylic acid:Initiator, and the MBA addition to this was varied and optimized as described below.

### 2.2. Battery Preparation

The cathode comprised electrolytic manganese dioxide (EMD, γ-MnO_2_) at 55 wt.%, carbon nanotubes (CNTs) at 35 wt.%, and bismuth oxide at 10 wt.%, the same as proposed by Ref. [9]. Each component was ball-milled together for 1 h. EMD was purchased from Borman (Henderson, NV, USA). CNTs were purchased from Cnano Technology Ltd., (Santa Clara, CA, USA), and bismuth oxide was purchased from Sigma Aldrich. The ball-milled mix was wetted with deionized (DI) water and then hand-cast onto a Cu-Ni current collector; then, the cathode was sealed with pellon and cellophane and pressed until the desired thickness of ~0.035 inches. Each current collector held ~23 mg of copper per cm^2^. Commercial sintered nickel (NiOOH) electrodes were used and purchased from Jiangsu Highstar Battery Manufacturing (Qidong, China). The size of the anodes and cathodes was 2.54 cm × 2.54 cm for the cyclic voltammetry (CV) and galvanostatic experiments, and was 5.08 cm × 7.62 cm for Zn-MnO_2_ full-cell cycling. Anodes and cathodes were assembled into a polysulfone box (8.255 cm width × 5.3975 cm depth × 15.875 cm height) and compressed with polypropylene shims. Then, the box was filled with 75 mL electrolyte on average. All cells were under vacuum for 30 min to soak the porous Zn and MnO_2_ electrodes. Mercury mercuric oxide (Hg-HgO) reference electrodes were used in each cell box to track half-cell voltages.

### 2.3. Electrolyte Spillability Safety Measurements

Battery “spillability” was measured according to U.S. DOT rules that declare the electrolyte is non-spillable if it does not flow through cracks or rupture in a battery case. Since commercial battery cases are packed tight with electrode stacks, creating typical flow gaps of ~1 mm, we measured spillability as the flow from 2.4 mm I.D. glass capillary tubes. Once 30 mm of hydrogel electrolyte was set up inside the end of these capillary tubes; each tube was dropped 10 mm end-first, under their own gravity, onto a hard surface to provide force promoting the electrolyte to flow out. Electrolyte flow out was recorded for different hydrogel cross-linking formulations. Additional hydrogel flow measurements were recorded when 100 mL of hydrogel fabricated in the bottom of a 250 mL beaker (diameter 75 mm) was tipped over to provide a force promoting flow; however, these measurements were not important for defining spillability according to US DOT regulations, because ruptured batteries have flow from capillary length scales much smaller than 75 mm.

### 2.4. Electrochemical Measurements

Cyclic voltammetry (CV) and electrochemical impedance spectroscopy (EIS) were performed through Biologic potentiostat/galvanostat (VSP Modular 5-channel). A multi-channel Arbin BT 2000 was used for galvanostatic experiments. After cycling, electrodes were removed, washed, and then soaked in DI water for 6 h and dried overnight in the air dryer at 50 °C. Scanning electron microscopy (SEM) and energy-dispersive X-ray spectrometry (EDX, EDS) were performed. SEM and EDX were performed by a FEI Helios Nanolab 660 Dualbeam FIB-SEM Operation fitted with an EDX (Thermo Fisher Scientific, Waltham, MA, USA).

### 2.5. Cu Diffusion Coefficient

Glass cuvettes (3.5 cm width × 1 cm depth × 4.5 cm height) were filled with either liquid or gel electrolytes that contained a known concentration of copper hydroxide (Cu(OH)_2_, from Alfa Aesar, Haverhill, MA, USA); see Figure S1. At the start of an experiment, a cuvette containing 1 molar Cu(OH)_2_ was fixed as the bottom cuvette. Next, in the case of hydrogel experiments, a cuvette with zero molar Cu(OH)_2_ hydrogel electrolyte was quickly fixed upside down on top of the first cuvette, creating a “step function” in Cu^2+^ concentration at the initiation of the experiment, as clearly seen by the sharp step in blue color in Figure S1. In the case of a liquid electrolyte experiment, an empty cuvette was fixed upside down on top of the bottom cuvette, and the liquid electrolyte with zero molar Cu(OH)_2_ was filled into the top cuvette via a hole drilled through the cuvette at its top. Photographs were taken on an hourly basis. The concentration profile of Cu^2+^ ions was calculated “colorimetrically” by use of the Beer–Lambert law, as explained in the Supplemental Information near Figure S1. Due to the precision shape of the glass cuvettes, there was no leakage between the cuvettes over the experiment. The experiments were conducted for 120 h.

## 3. Results and Discussion

### 3.1. Non-Spillable Hydrogel Experiment

To find the optimal hydrogel cross-linker formulation, the US DOT spillability methods described above were repeated on hydrogels with varying amounts of cross-linker. More cross-linker causes a higher overvoltage during cycling, so less cross-linker was preferred. We settled on a hydrogel formulation that had the smallest amount of cross-linker but kept zero flow from the 2.4 mm glass capillary experiments. In other words, we kept the electrolyte “non-spillable” but otherwise minimized hydrogel cross-linking (data shown in Table 1). As the data of Table 1 show, a 3.92 × 10^−5^ mole fraction of MBA was determined as the optimal cross-linked hydrogel. All the tested hydrogels in Table 1 retained their initial rheology over the time studied.

**Figure 1:**
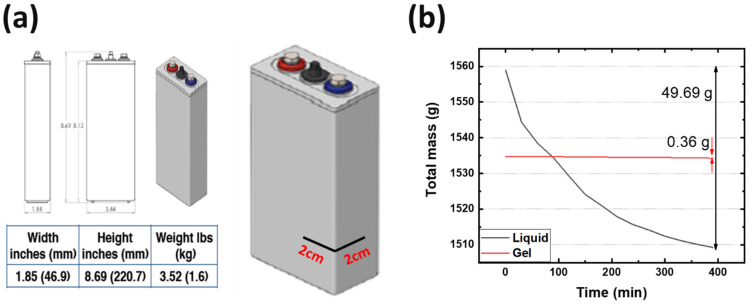
(**a**) A manufactured prismatic cell information and (**b**) time vs. total mass change after making cracks.

Using the optimized hydrogel formulation from Table 1, the optimized hydrogel electrolyte was tested for spillability from a real battery cell manufactured by Urban Electric Power (UEP), as described in Figure 1a. Figure S2 shows the electrode stack inside the cell box, which confirms the ~2 mm capillary length scales of our spillability methods. The UEP manufactured batteries were filled with liquid and gel electrolyte separately up to the top of the electrodes. Cuts in the cell box were intentionally made using a razor blade, at locations 2 cm height from the bottom of the cells. The width of each cut was 2 cm, as shown in Figure 1a. Due to gravitational force, the electrolyte attempted to flow from the cracks. Any electrolyte emerging from the cracks was wiped away with paper towels, and the total weight of the batteries was measured every 30 min for several hours. Mass loss is due to leakage of electrolyte through the cut to the case. Figure 1b shows the resulting data, wherein the cell with liquid electrolyte had 49.69 g of mass loss, while the cell with gel electrolyte had only 0.36 g of mass loss. Therefore, the hydrogel electrolyte was determined to satisfy the DOT regulation for non-spillable batteries.

### 3.2. Electrochemical Performance

**Figure 2:**
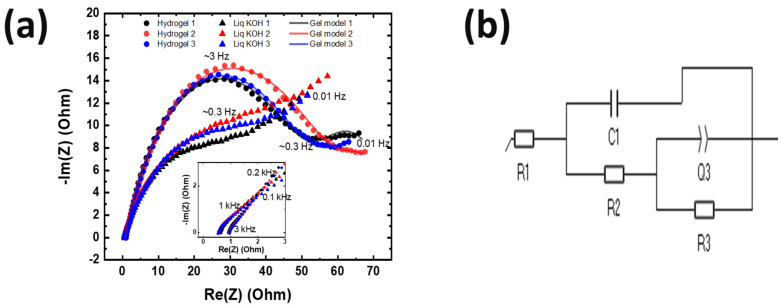
(**a**) Cell impedance measurement of Zn plate symmetric cell with the gel electrolyte and a liquid electrolyte. The experiment was conducted three times for each electrolyte. Transparent data points are the fit. (**b**) The equivalent circuit model from the fits in (**a**). R1 is the solution resistance; R2 is the charge-transfer resistance at Zn and electrolyte interface; R3 is the resistance associated with OH^−^ transport; C1 is the capacitance; Q3 is the constant phase element for non-faradaic charging of the double layer.

The non-spillable optimized hydrogel electrolyte was then used in measurements of Zn^2+^ and Cu^2+^ ion diffusion. To understand Zn^2+^ diffusion behavior, two Zn foil symmetric electrodes were used (as shown in Figure S3) for EIS measurements, which were repeated three times with liquid and gel electrolyte. The EIS scanning frequency range was 100 kHz to 0.01 Hz. As shown in Figure 2a, due to the viscous properties and polymer chain of the gel electrolyte, the solution resistance of the gel electrolyte was ~0.4 Ohm higher than the liquid electrolyte, and the curve had a deeper sigmoidal shape. In the low-frequency range, the plots of the liquid electrolyte had a slope close to 45 degrees, whereas the gel electrolyte plots showed a lower slope. This suggests that the zinc transfer rate in the gel electrolyte is slower than in the liquid electrolyte but not negligible, as evidenced by the plotted slope. The ionic conductivity was calculated by Equation (5):

(5) $$\sigma =\left(\frac{1}{R}\right)*\left(\frac{l}{A}\right)$$

where *R* is charge-transfer resistance, *l* is thickness, and *A* is area [24]. From Figure 2a, the value of charge-transfer resistance was obtained, and the ionic conductivity of Zn with the gel electrolyte was ~5.45 mS/cm. One of the recent studies reported that their hydrogel electrolyte had 83 mS/cm [25]. Even though their components are similar to our gel electrolytes, the difference could be due to soaking their hydrogel electrolyte in ZnSO_4_ solutions overnight, so that additional species such as sulfate ions helped obtain higher ionic conductivity than ours. Moreover, Figure S12 of Ref. [25] showed that their gel electrolyte was solid-like. The phase difference also made the difference.

**Figure 3:**
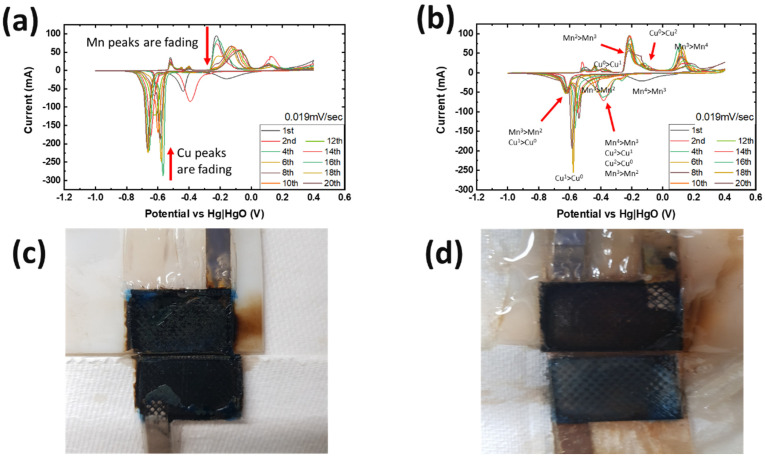
The CV experiments were conducted at 0.019 mV/s, which means it takes 20 h to charge and discharge. The CV results with (**a**) liquid electrolyte and (**b**) gel electrolyte. The picture of the dissected cell 12th cycle after galvanostatic with (**c**) liquid and (**d**) gel electrolyte.

Figure 3a,b present CV plots with liquid and gel electrolyte, respectively. With liquid electrolyte, the Mn^3+^ to Mn^2+^ peaks at −0.7 V vs. Hg-HgO were sharper than with gel. As this voltage range is for the dissolution–precipitation reaction, dissolved Mn ions were intercalated with dissolved Cu ions, leading to Cu^2+^-intercalated Bi-birnessite [9]. However, as the Cu^1+^ to Cu^0^ peak at −0.6 V vs. Hg-HgO started fading at the 12th cycle, it suggested that dissolved Cu ions were not able to react with Mn ions during the cycle. Cu and Mn peaks at the same voltage were stronger than with hydrogel electrolyte until the 20th cycle. In the oxidation portion, the gel electrolyte cell showed Mn peaks at −0.2 and 0.2 vs. Hg-HgO for all cycles, stronger than for liquid electrolyte, especially after the first few cycles. This is because hydrogel electrolyte limited the dissolution of active materials, and thereby mitigated active ion loss.

**Figure 4:**
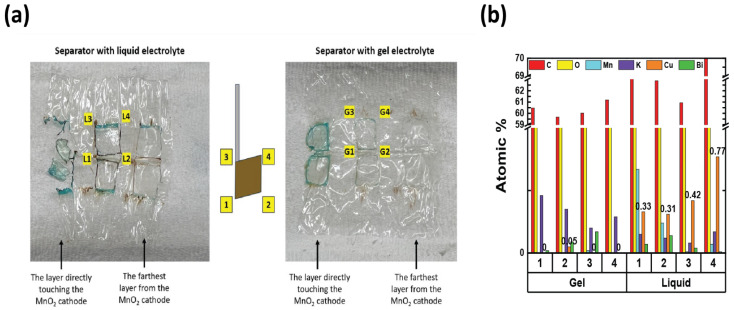
(**a**) SEM/EDX analysis was conducted on the separator at the four corners where the electrode was as shown in the electrode scheme in the middle. 1 and 2 represent the bottom two corners, and 3 and 4 represent the top two corners. L and G mean liquid and gel electrolyte, respectively. (**b**) The atomic % of six species from the four corners of the separators in (**a**).

To investigate the reason for the CV measurement, with liquid electrolyte showing the Cu peak reduced after the 12th cycle, two identical cells with MnO_2_ vs. NiOOH electrodes at C/20 (capacity of MnO_2_) were built, one with liquid KOH and the other with hydrogel KOH, and both were cycled until the 1st, 5th, or 12th cycle and dissected. The blue color seen in the dissected materials (see Figure 3c,d and Table 2) is due to Cu^2+^ ions. This increased migration of blue-colored Cu^2+^ in cells with liquid KOH correlates with more metallic-colored Cu deposited on the separators of cells with liquid electrolyte; see Table 2 and Figure S4. But with hydrogel electrolyte, this deposition of Cu ions was limited and more localized in the electrode area. After the first cycle with liquid electrolyte, the deposition of Cu ions was observed on the second layer of the separator. After the fifth cycle, the separator was observed to be degraded and the blue color was significantly denser than in the experiment with hydrogel electrolyte. To quantify Cu ions from the separators, identical experiments conducted as in Table 2 were carried out. The amount of cathode materials and the dimension of the electrodes were proportionally scaled down to fit 1.27 cm × 1.27 cm and then dissected after their cycling. The cells were cycled until the fifth cycle so that the separator would retain structural integrity, as shown in Table 2, enabling the analysis of the blue color in separators for both electrolytes. As specifically described in Figure 4a, SEM/EDX analysis was conducted at the four corners, and the EDX mapping results are shown in Figures S5–S8. The mapping analysis detected six species corresponding to the initial battery species, and elemental Cu, one of the six, was diffused in the separators of both liquid and gel electrolyte. However, it is noted that, in Figure 4b, the corners of the gel electrolyte separator detected less than 0.05 atomic % Cu, while the separator of cycled liquid electrolyte had detected 6–15 atomic % times higher Cu. This supports the results from Figure 3 and Figure S4 and Table 2 and agrees that hydrogel electrolytes mitigated Cu ion diffusion while cycling.

**Figure 5:**
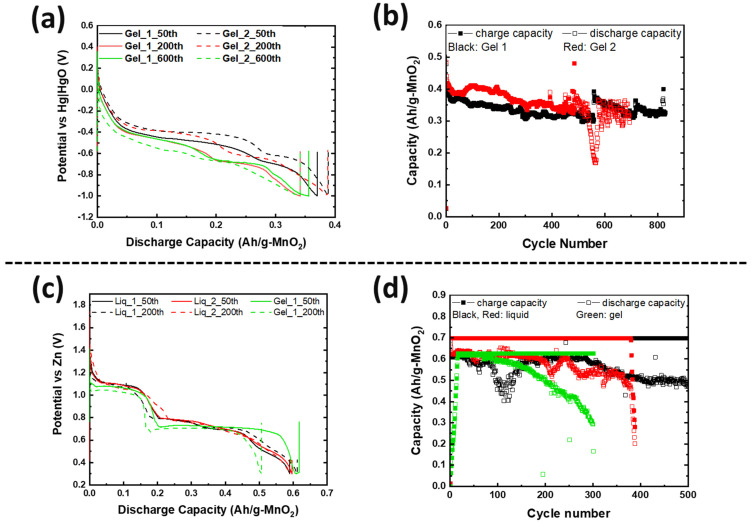
(**a**) The galvanostatic data of identical MnO_2_ half cells against NiOOH with gel electrolytes at C/3, where C is based on 2-electron MnO_2_ capacity. (**b**) Capacity retention of the MnO_2_ cells from (**a**). (**c**) The galvanostatic data of full cells with liquid and gel electrolytes at C/20, where C is based on 2-electron MnO_2_ capacity. (**d**) Capacity retention of the full cells from (**c**).

Figure 5a,b demonstrate galvanostatic cycling performance from two identical MnO_2_ half cells (vs. NiOOH) with gel electrolyte, which achieved ≥700 cycles with 99% coulombic efficiency and 63% energy efficiency. Our group reported [9] that the cycle life with liquid electrolyte is over 1000 cycles at the same cycling rate. Even though our present study of gelled KOH did not replicate the cycling performance reported in Ref. [9], our present results indicate optimistic outcomes, as we have observed that the gel electrolyte mitigated Cu diffusion so that it keeps MnO_2_ reversibility. In Figure 5c,d, the two liquid-containing cells achieved ~500 cycles, while the cell with gel electrolyte performed 300 cycles until it showed 50% theoretical capacity fade. This is because the gel-containing cell had a twice greater Zn utilization than the two liquid-containing cells. If the gel-containing cell had the same Zn utilization, it is hypothesized that its cycle life would be at least equal to the liquid-containing cell’s performance due to the mitigation of failure mechanisms caused by zinc. These electrochemical results indicated that the gel electrolyte helps reduce Cu ion loss so that active ions, such as Mn, Cu, and Bi, were able to react with each other, leading to the stable electrochemical reaction reversibility of the [(Cu-Bi)Mn] complex. In this way, using gel electrolytes will deliver a longer battery cycle life than cells with liquid electrolyte.

A reduction of Cu^2+^ diffusion was hypothesized to explain why copper migrates less in a cell with hydrogel electrolyte. To test this hypothesis, we measured the Cu^2+^ diffusion coefficient in liquid and gel electrolyte by fitting analytical solutions of Fick’s law to the data we collected on time-varying concentration of Cu^2+^ in our cuvette experiments; see Methods Section and Supplemental Information near Figure S1. Fick’s law holds

(6) $$\frac{\partial C}{\partial t}=D\frac{\partial^{2}C}{\partial y^{2}}$$

where *C* is the concentration of Cu, *t* is time, *D* is the diffusion coefficient, *y* is position. The cuvette experiments were homogenous in all directions except the y-direction. The boundary conditions (B.C.) and the initial conditions (I.C.) were experimentally, and analytically, as follows:

(7) $$B.C.\;\;\;\;\;\;\;\;\;\;\;\;\;\;\frac{\partial C}{\partial y}=0\;\;\;\;\;\;\;\;\;\;\;\;\;\;\;\;\;\;\;\;\;\;\;\;\mathrm{at}\;y=0\;\mathrm{and}\;L$$

(8) $$I.C.\;\;\;\;\;\;\;\;\;\;\;\;\;\;C\left(y,0\right)=\left\{\begin{array}{c} \;0,\;\;H<y<L\\ \;1,\;\;0<y<H \end{array}\right.\;\;\;\;\;\;\;\;\;\;\;\;\;\;\;\;\;\;\;\;\;\;\;\;\;\;\;\;\;\;\;\;\;\;\;\;\;\;\;\;\;\;\;\;\;\;\;\;\;\;\;\;$$

The analytical solution *C*(*y*,*t*) satisfies B.C., I.C., and the governing equation, and was determined by separation of variables, then Fourier series reconstruction. Using the eigenfunctions of this system, the entire solution is described as

(9) $$C_{n}\left(y,t\right)=A_{0}+\sum_{n=1}^{\infty }A_{n}\cos\frac{n\pi y}{L}e^{-\lambda_{n}^{2}t}\;\;\mathrm{and}\;\lambda_{n}=\sqrt{D}\frac{n\pi }{7}$$

The coefficients, *A*_0_ and *A_n_*, were determined by Fourier cosine series. In our experiments, *L* is 7 cm and *H* is 3.5 cm, so the final analytical solution is

(10) $$C\left(y,t\right)=\frac{1}{2}+\sum_{n=1}^{\infty }\frac{2}{n\pi }\sin\frac{n\pi }{2}\cos\frac{n\pi y}{7}e^{-\lambda_{n}^{2}t}\;\mathrm{and}\;\lambda_{n}=\sqrt{D}\frac{n\pi }{7}$$

**Figure 6:**
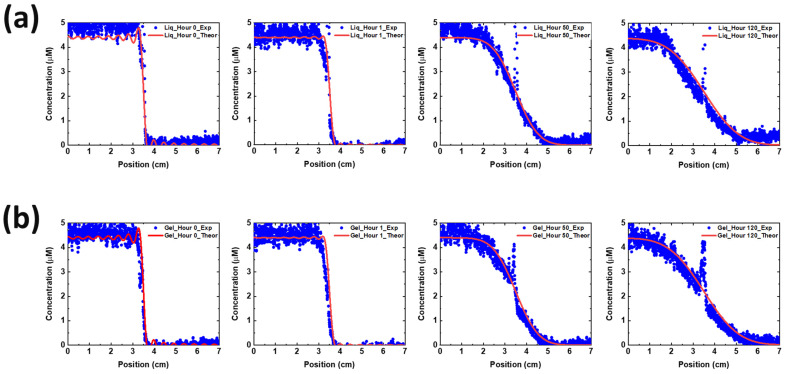
Cu diffusion quantification and plots with (**a**) liquid (top four figures in a row) and (**b**) gel electrolyte (bottom four figures in a row). The experiments were conducted for 120 h. Blue dots represent the experimental data and solid line represents the theoretical solution.

Using the first 30 terms of this Fourier series, the experimental data and analytical solution are overplotted in Figure 6. The diffusion coefficient was determined by fitting the experimental and theoretical data, with D=1.9×10−6 cm2s being the best fit. We find the same diffusion coefficient in liquid and hydrogel electrolytes to within and experimental error of ~0.3×10−6 cm2s, which in retrospect is less surprising when considering the volume fraction of water in the hydrogel is 95%. The spike in data points near the middle (near *y* = 3.5 cm) of the plots in Figure 6 have a high experimental error due to this location being where the two cuvettes touched, which caused optical refraction due to the glass edges.

Since Cu^2+^ ion diffusion on the molecular level appears to be the same in gel and liquid KOH electrolyte, we must hypothesize a different explanation for the dissected battery cells showing reduced Cu migration. We speculate that gel electrolyte reduced convection of the electrolyte, which is forced by several factors (electrical, expansion/shrinkage cycles, bubble growth) [26,27]. This can be supported with Figure S9. The pictures in Figure S9 were taken while charging. As shown, bubbles went upwards and were removed from the same spot where they generated. They did not go anywhere in the electrolyte, supporting the speculation of reduced convection by gel electrolyte. Further, the separators with gel electrolyte in Table 2 showed light reflection on each layer of separators. This means that the gel electrolyte was evenly applied during the vacuum process. With this, we hypothesize that gel electrolyte leaves a thin film between the separators that can reduce the direct contact of conductive Cu depositions on the separator, thus reducing short circuit severity or frequency. Further studies will be needed to confirm these hypotheses.

## 4. Conclusions

Cross-linked hydrogel electrolytes were optimized to pass the US Department of Transportation guidelines for non-spillable batteries to allow transportability. This was achieved by investigating electrolyte flow from cracks or ruptures to the battery cell box. The optimized gel electrolyte was then studied for the second electron Mn cathode reaction. A series of electrochemical experiments with the hydrogel electrolyte showed a successful electrochemical reaction of each active material under the second electron reaction chemistry. The CV experiments suggested that the gel electrolyte helped stabilize the Cu-Bi Mn complex, helping the reversibility of Bi-birnessite and therefore the long-term cycle life of the battery. Dissected cells showed less Cu^2+^ migration in gel electrolyte as compared to liquid electrolyte. The Cu^2+^ diffusion coefficient was measured to be the same in liquid and gel electrolyte, and so we suggest the reason for the reduced Cu^2+^ migration is that hydrogel electrolyte reduces convection and also provides a non-conductive physical barrier between conductive materials and the separator itself. This study supports the optimism that gel electrolytes could be applied to long-duration energy storage applications, continuously providing a second electron Mn cathode reaction and a two-electron Zn anode reaction.

## Figures and Tables

**Table 1:** Hydrogel flow measurement used to optimize hydrogel formulation. Mole fraction of MBA to H_2_O is the first column. Cross-linking was allowed to proceed for at least 16 h prior to experiments.

Mole Fraction MBA:H_2_O	Flow from ~1 mm Gap	Flow from ~75 mm Gap
2.61 × 10^−5^	Flow	Flow
3.40 × 10^−5^	Flow	Flow
3.92 × 10^−5^	No Flow	Flow
4.70 × 10^−5^	No Flow	Flow
5.20 × 10^−5^	No Flow	Flow
6.00 × 10^−5^	No Flow	Flow
6.50 × 10^−5^	No Flow	No Flow
7.30 × 10^−5^	No Flow	No Flow
7.80 × 10^−5^	No Flow	No Flow

**Table 2:** Separators from dissected cells after C/20 galvanostatic experiments.

	1st Cycle	5th Cycle	12th Cycle
Liquid KOH Electrolyte	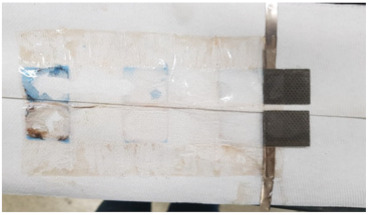	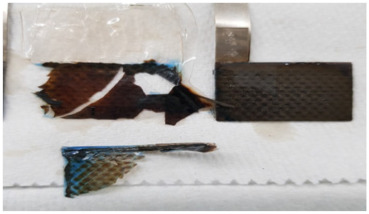	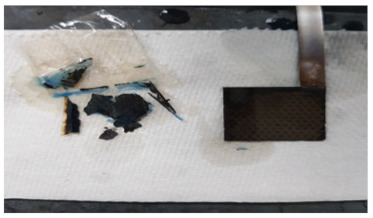
Gel Electrolyte	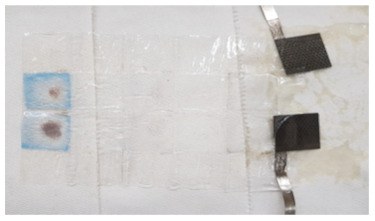	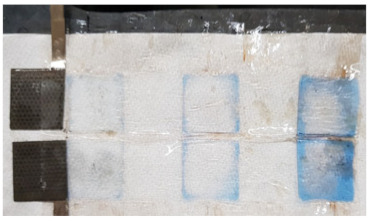	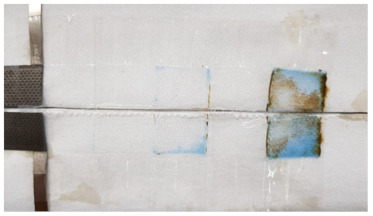

## Data Availability

The data presented in this study are available on request from the corresponding author.

## Supplementary Materials

The following supporting information can be downloaded at: https://www.mdpi.com/article/10.3390/polym16050658/s1, Figure S1: Cu diffusion coefficient measured by colorimetry applied to diffusion of Cu(OH)_2_ from bottom cuvette into top inverted cuvette, photos taken at (a) the beginning of an experiment, and (b) 120 hours afterwards. The four cuvettes sitting on the table on the left hold reference concentrations of 0, 0.3, 0.6 and 1 molarity; Figure S2: The top view of a prismatic cell and the information of the gap between the electrodes and left and right side of the cell box; Figure S3: The view of Zn plate symmetric experiment; Figure S4: Identical cell construction after the 60th cycle with liquid and gel electrolyte cycled at C/20 where C is 2-electron MnO_2_ capacity; Figure S5: SEM images and EDX mapping results from the four corners of the separator with liquid electrolyte. All scale bars are 500 μm; Figure S6: SEM images and EDX mapping results from the four corners of the separator with gel electrolyte. All scale bars are 500 μm; Figure S7: The peak intensity results for the 6 species with liquid electrolyte; Figure S8: The peak intensity results for the 6 species with gel electrolyte; Figure S9: Bubble behavior in the gel electrolyte while charging; Table S1: Molar absorptivity of reference cuvettes.
