# Supplementary material for: Use of Hydrogel Electrolyte in Zn-MnO2 Rechargeable Batteries: Characterization of Safety, Performance, and Cu2+ Ion Diffusion
Source: Polymers (Basel). 2024 Feb 28;16(5):658. doi: 10.3390/polym16050658 (PMC10934817; doi:10.3390/polym16050658)
Supplement: Supplementary file 1 [file polymers-16-00658-s001.zip › polymers-2829272-supplementary.pdf]

Supplementary Information

Use of Hydrogel Electrolyte in Zn-MnO_2 Rechargeable Batteries: Characterization of Safety, Performance, and Cu^{2+} Ion Diffusion

Jungsang Cho [1], Damon E. Turney [1,*], Gautam Ganapati Yadav [2], Michael Nyce [1], Bryan R. Wygant [3], Timothy N. Lambert [3,4] and Sanjoy Banerjee [1]

[1] The CUNY Energy Institute, City University of New York, 160 Convent Ave, New York, NY 10031, USA; chojs0114@gmail.com (J.C.); mnyce1957@gmail.com (M.N.); sanjoy@urbanelectricpower.com (S.B.)

[2] Urban Electric Power, Pearl River, NY 10965, USA; gautam@urbanelectricpower.com

[3] Sandia National Laboratories, Department of Photovoltaics and Materials Technology, Albuquerque, NM 87185, USA; bwygant@sandia.gov (B.R.W.); tnlambe@sandia.gov (T.N.L.)

[4] Sandia National Laboratories, Center of Integrated Nanotechnologies, Albuquerque, NM 87185, USA

* Correspondence: dturney@ccny.cuny.edu

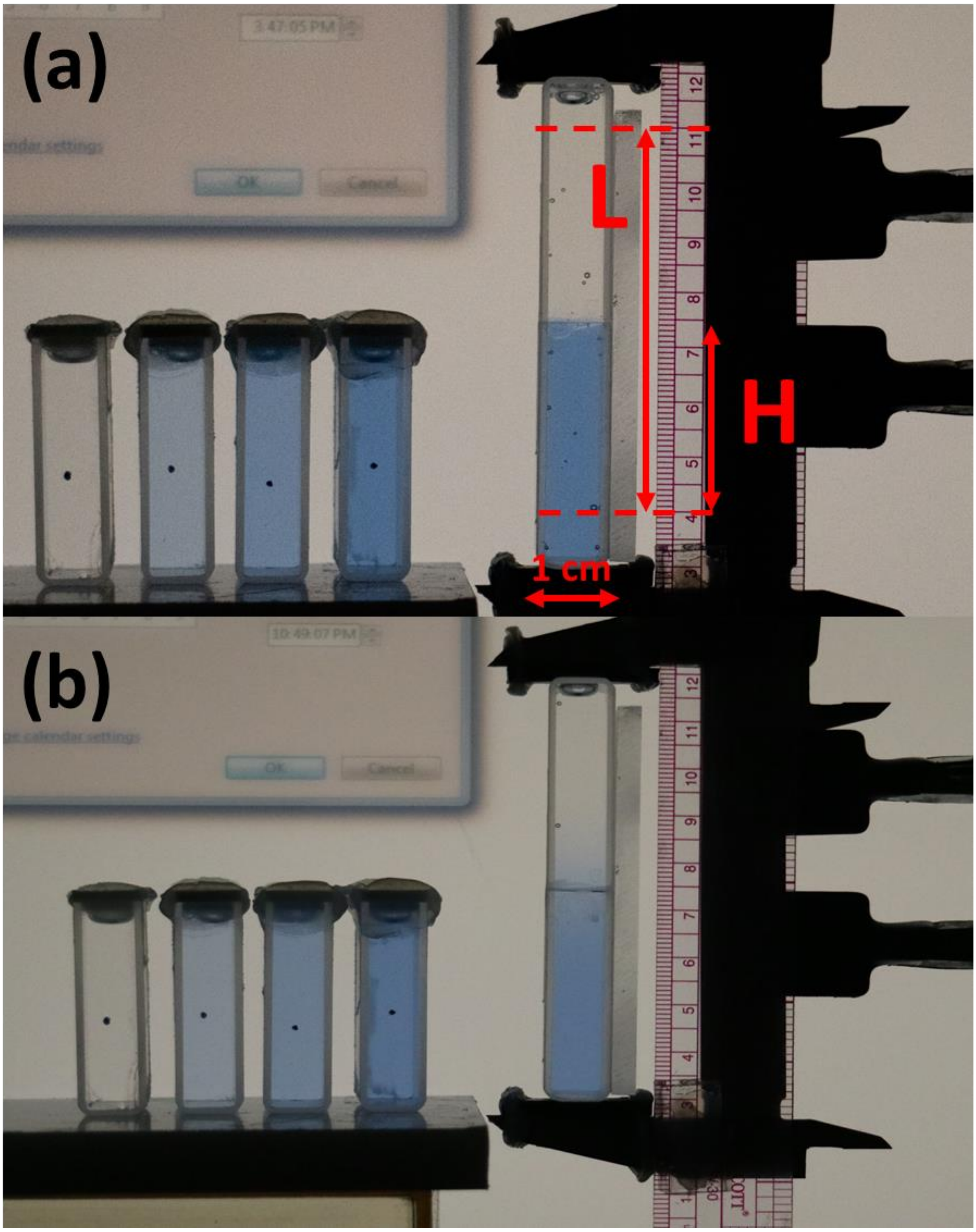

Figure S1. Cu diffusion coefficient measured by colorimetry applied to diffusion of $Cu(OH)_2$ from bottom cuvette into top inverted cuvette, photos taken at (a) the beginning of an experiment, and (b) 120 hours afterwards. The four cuvettes sitting on the table on the left hold reference concentrations of 0, 0.3, 0.6 and 1 molarity.

From the reference cuvettes of Fig S1, absorption of the light was calculated via Beer-Lambert law as described:

$$\ln\left(\frac{I_0}{I}\right) = A = \varepsilon\, l\, c \text{ and } \varepsilon = \frac{-\ln\left({}^{I}/_{I_0}\right)}{c \times l}$$

where A: absorbance, I: transmitted light, I_0: incident light, c: concentration (M, moles/mL), l: length of light path (cm) and ε: molar

absorptivity ($M^{-1}cm^{-1}$). The value of I_0 was taken from the cuvette with zero Cu mole fraction. As shown in Table S1, the average value of molar absorptivity was determined as 0.04239.

Table S1. Molar absorptivity of reference cuvettes.

	Cuvette 1 (I_0)	Cuvette 2	Cuvette 3	Cuvette 4
Molar absorptivity	-	0.04185	0.04302	0.04231

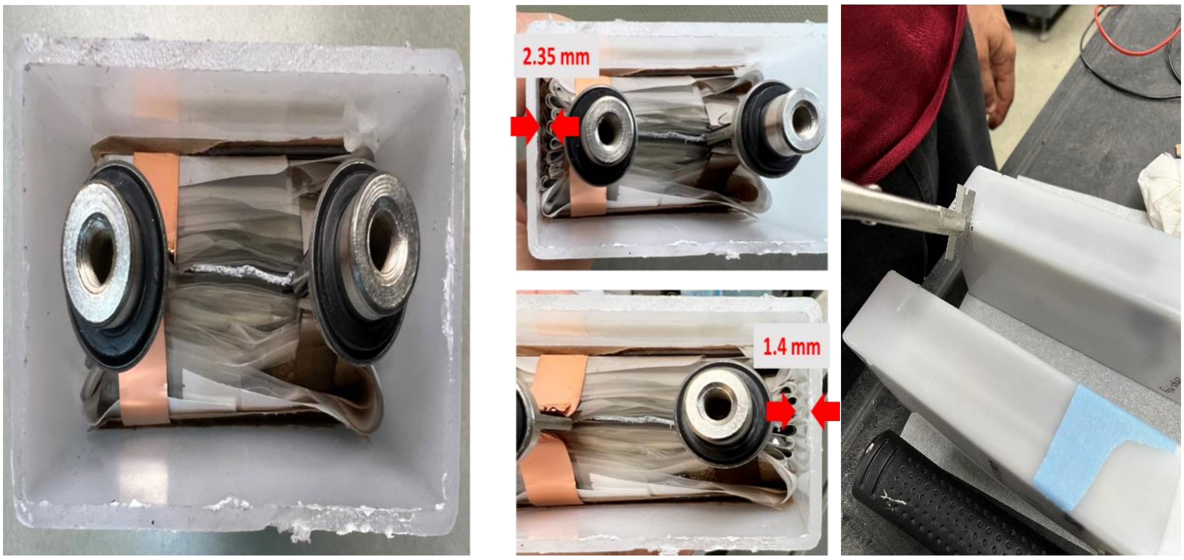

Figure S2. The top view of a prismatic cell and the information of the gap between the electrodes and left and right side of the cell box.

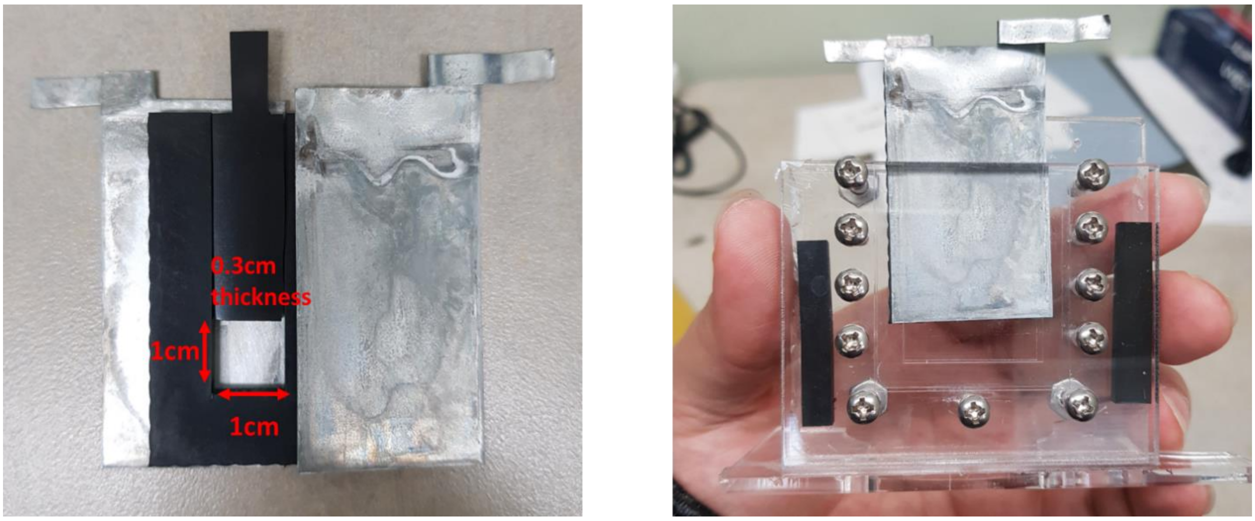

Figure S3. The view of Zn plate symmetric experiment.

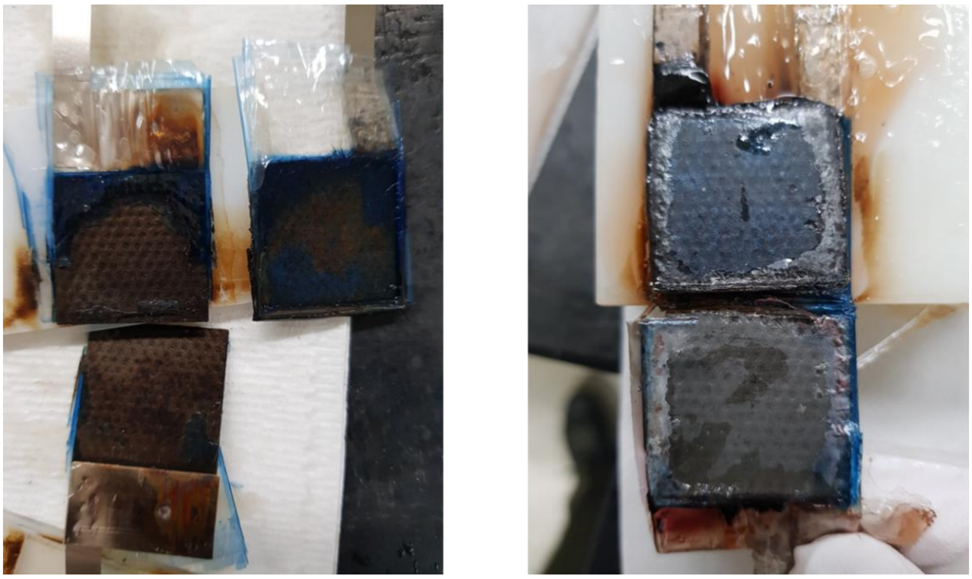

Figure S4. Identical cell construction after the 60th cycle with liquid and gel electrolyte cycled at C/20 where C is 2-electron MnO_2 capacity.

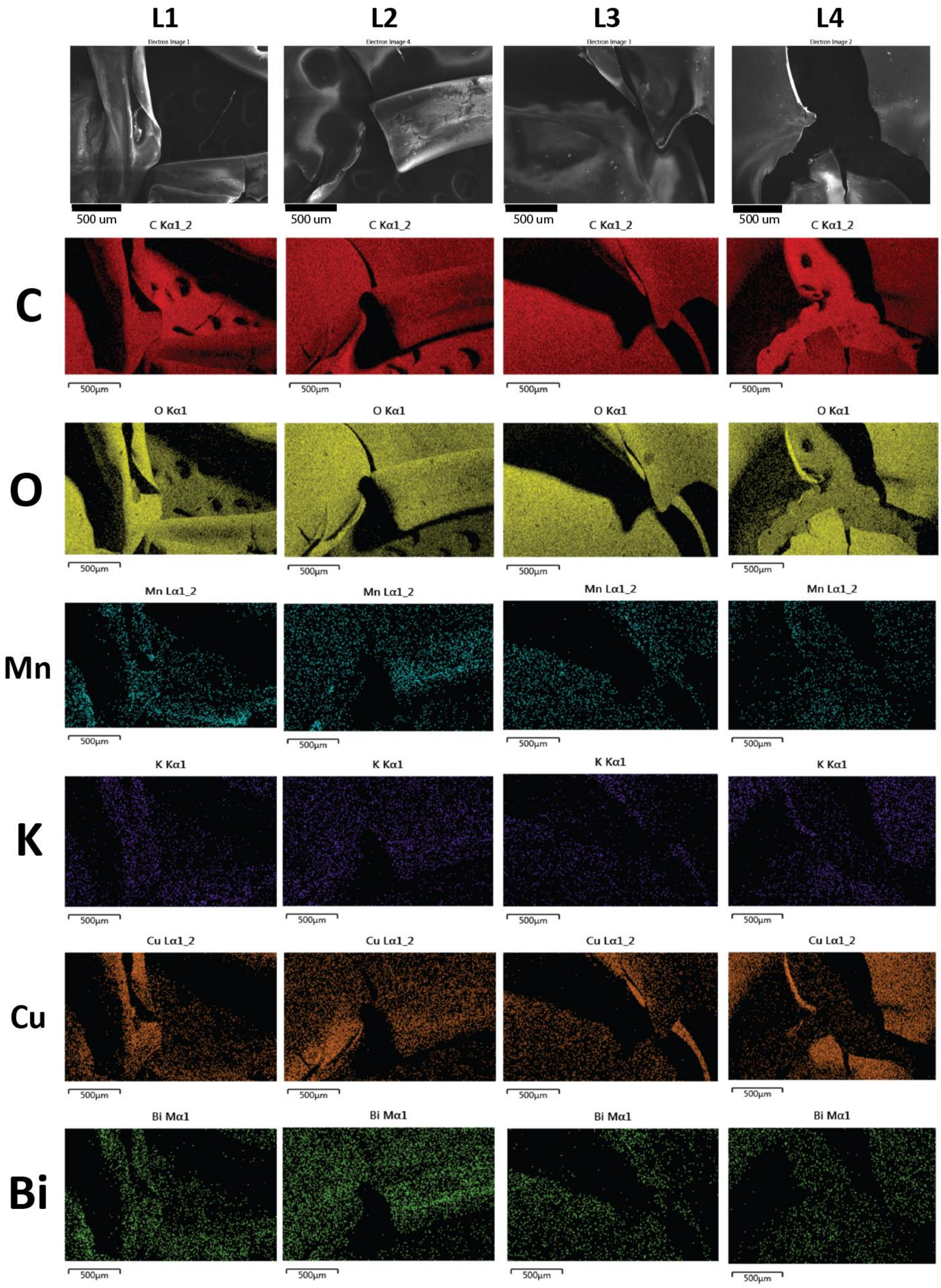

Figure S5. SEM images and EDX mapping results from the four corners of the separator with liquid electrolyte. All scale bars are 500 μm.

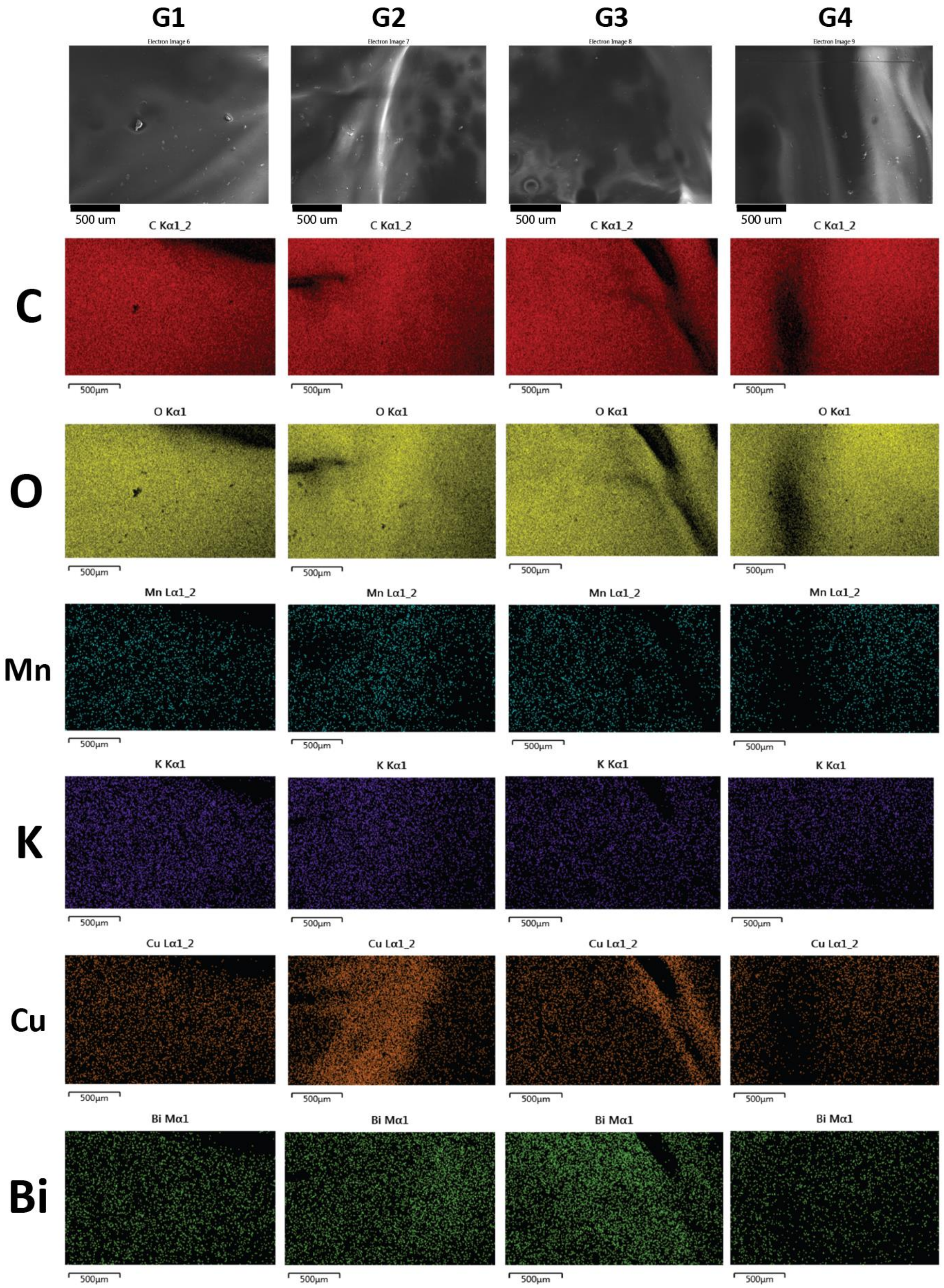

Figure S6. SEM images and EDX mapping results from the four corners of the separator with gel electrolyte. All scale bars are $500\ \mu m$.

Liquid

1

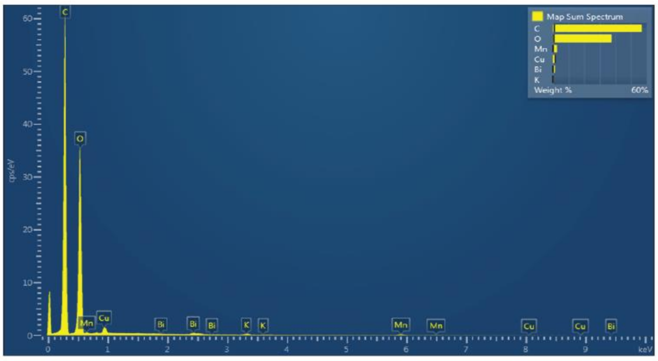

2

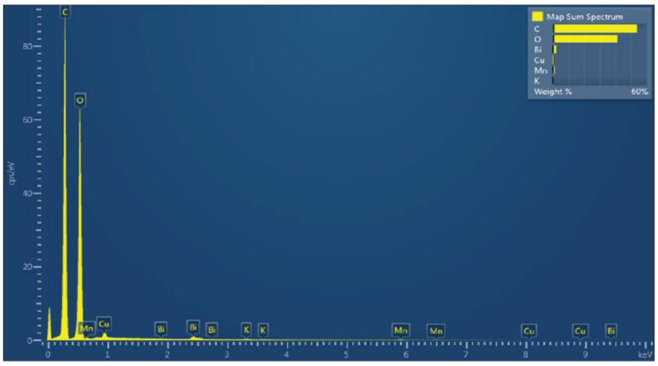

3

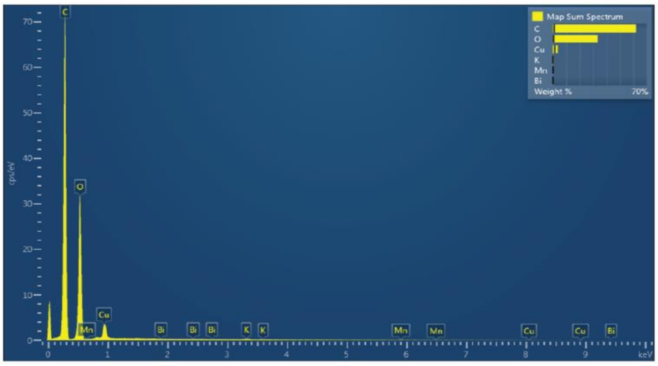

4

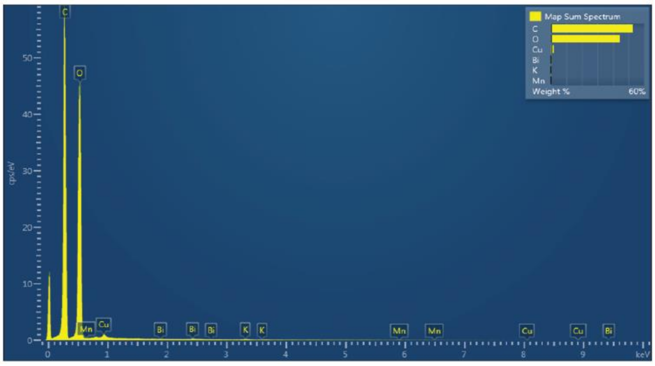

Figure S7. The peak intensity results for the 6 species with liquid electrolyte.

Gel

1

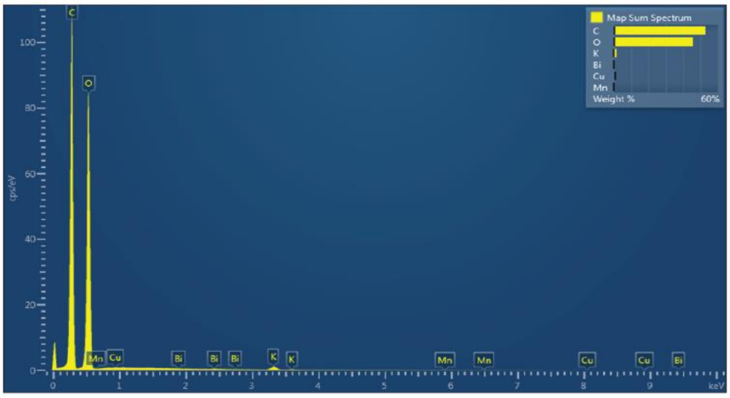

2

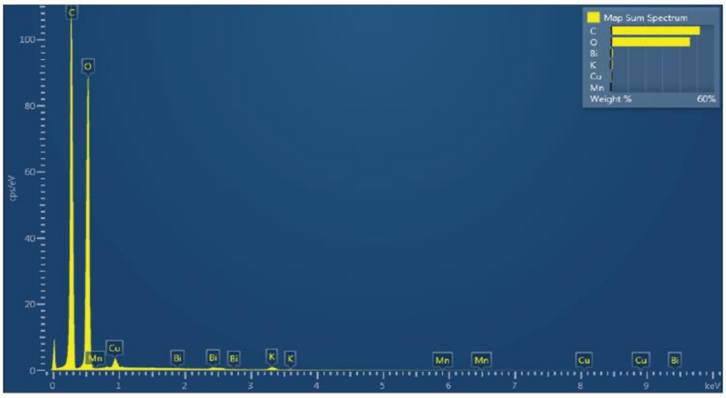

3

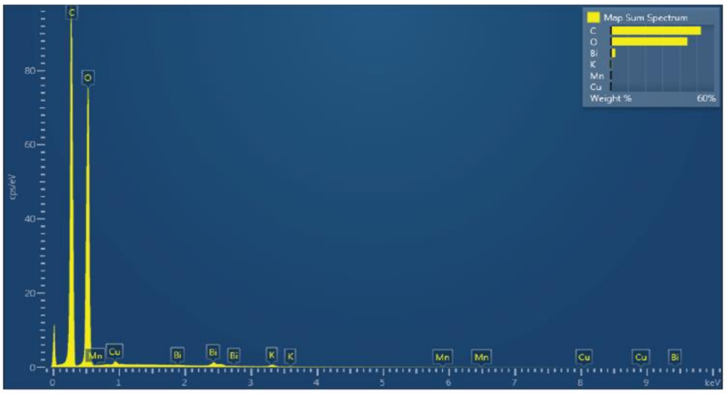

4

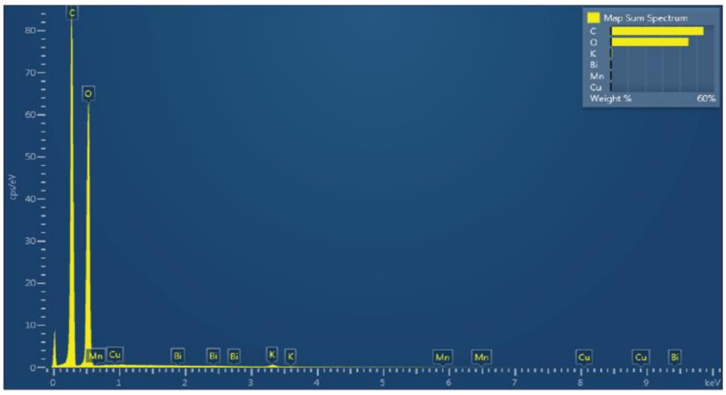

Figure S8. The peak intensity results for the 6 species with gel electrolyte.

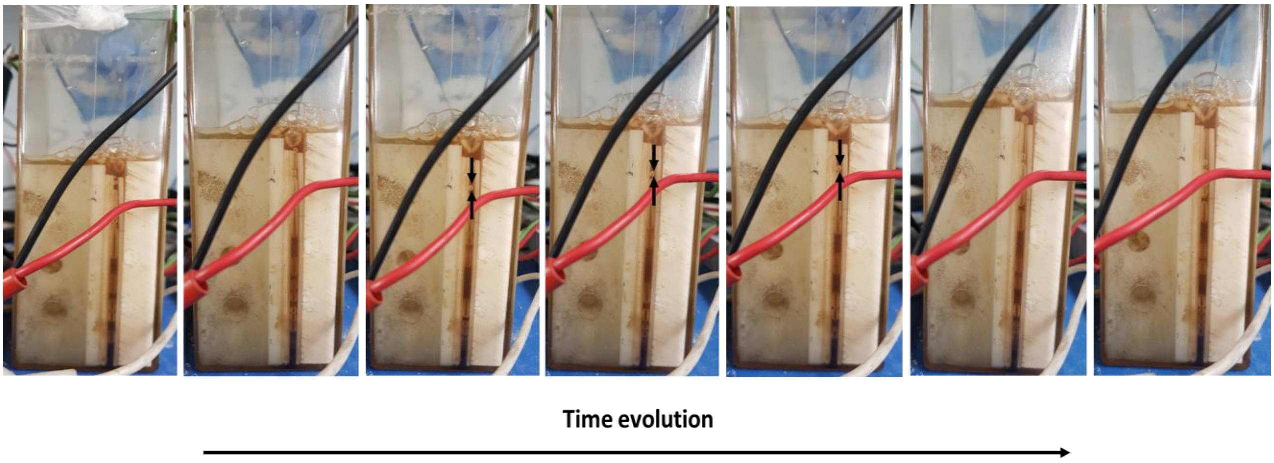

Figure S9. Bubble behavior in the gel electrolyte while charging.
